# Sociodemographic characteristics are associated with prevalence of high-risk waist circumference and high-risk waist-to-height ratio in U.S. adolescents

**DOI:** 10.1186/s12887-021-02685-1

**Published:** 2021-05-03

**Authors:** Felicia J. Setiono, Laura A. Guerra, Cindy Leung, Tashara M. Leak

**Affiliations:** 1 Division of Nutritional Sciences, Cornell University, Ithaca, NY 14853 USA; 2 Department of Health & Behavior Studies, Teachers College, Columbia University, New York, NY USA; 3 Department of Nutritional Sciences, University of Michigan School of Public Health, Ann Arbor, MI USA

**Keywords:** Waist circumference, Waist-to-height ratio, Adolescents, Abdominal obesity, Adiposity

## Abstract

**Background:**

Adiposity status in adolescence is associated with various health outcomes in adulthood. Waist circumference [WC] and waist-to-height ratio [WHtR] are measures of abdominal obesity and have shown to be valid predictors of future chronic diseases. However, the relationship between sociodemographic characteristics and WC, as well as WHtR in U.S. adolescents remain unclear. Thus, the study aims to examine associations between sociodemographic characteristics and abdominal obesity among a nationally representative sample of U.S. adolescents.

**Methods:**

The sample included 4712 adolescents (12–19 years) in the 2007–2016 National Health and Nutrition Examination Survey. Associations between sociodemographic characteristics and abdominal obesity (WC and WHtR) were examined using multiple logistic regression models, adjusted for age, physical activity level, and sedentary activity.

**Results:**

Around 18% of adolescents had high-risk WC (≥90th percentile) and 34% had high-risk WHtR (≥0.5). Females had higher odds of high-risk WHtR compared to males (OR = 1.46, 95%CI = 1.23–1.72). Mexican American adolescents had higher odds of high-risk WHtR compared to non-Hispanic White (OR = 1.66, 95%CI = 1.24–2.20), non-Hispanic Black (OR = 1.73, 95%CI = 1.26–2.36), and other race/multi-racial adolescents (OR = 1.84, 95%CI = 1.21–2.80). When their parent were college graduates, adolescents had lower odds for high-risk WC compared to when the parent had some college education (OR = 0.68, 95%CI = 0.49–0.93) or a high school degree or less (OR = 0.70, 95%CI = 0.51–0.97). Similar associations were seen between parental education level and high-risk WHtR, as well as between household income and high-risk WHtR.

**Conclusions:**

Measures of abdominal obesity should be considered to assess burden of adiposity, especially among female adolescents, adolescents from racial/ethnic minority and low socioeconomic status backgrounds. Additionally, future health interventions should consider including changes in WC and WHtR to measure the impact of these interventions.

## Background

Obesity during adolescence is a risk factor for various chronic diseases in adulthood [1–3]. The most common measure of obesity in adolescents is body mass index (BMI), where a BMI exceeding the age- and sex- specific 95th percentile (equivalent to BMI z-score of 1.64) indicates obesity (based on the 2000 Centers for Disease Control and Prevention (CDC) BMI-for-age growth charts) [4, 5]. In the U.S., adolescents (12–19 years) experience a greater prevalence of obesity compared to younger children. Using data from National Health and Nutrition Examination Survey (NHANES) from 2013 to 2016, Ogden et al. reported the prevalence of obesity in adolescents is 20.6% (95%CI = 17.6–23.8) [6]. Additionally, it is known that sociodemographic characteristics are associated with obesity prevalence; adolescents from racial/ethnic minority and low socioeconomic status (SES) households experience higher prevalence of obesity than their non-Hispanic white and higher SES counterparts [6–9].

While BMI is often used as an indicator for adiposity, it is often criticized because it does not differentiate between lean mass and fat [10]. In contrast, waist circumference (WC) and waist-to-height ratio (WHtR) are measures of abdominal obesity and are shown to be better predictors of cardiovascular disease risk factors, type 2 diabetes, and metabolic syndrome [11–16]. Despite the value of these measures, studies examining adolescents’ WC and WHtR are sparse and less is known about sociodemographic disparities in WC and WHtR among U.S. adolescents. Using 2003–2012 NHANES data, Xi and colleagues found that female adolescents have a higher prevalence of high-risk WC and WHtR compared to males, and that Mexican American adolescents have a significantly higher prevalence of high-risk WC and WHtR compared to their non-Hispanic white counterparts [11]. Research suggests that higher parental education level and household income are associated with lower prevalence of BMI among U.S. adolescents [6, 17, 18], but the relationship between these SES factors and WC, as well as WHtR, among U.S. adolescents in recent years remains unknown. Using more recently available data from 2007 to 16 NHANES, the overall aim of this study was to examine, 1) the prevalence of high-risk WC (≥90th percentile) and WHtR (≥0.5), and 2) associations between sociodemographic characteristics and high-risk WC and WHtR, among a nationally representative sample of U.S. adolescents (12–19 years).

## Methods

### Study design and population

The NHANES is a complex, multistage probability cross-sectional study of a nationally representative sample of U.S. civilian, noninstitutionalized populations [19]. The National Center for Health Statistics within CDC conducts the NHANES in two-year cycles. For the current study, five NHANES cycles, 2007–2016, were included in the analyses. A total of 5795 adolescents (12–19 years) participated in NHANES 2007–2016 and 4712 were included in the analyses with complete sociodemographic, WC, and WHtR data. Adolescents 12–17 years provided assent and their parents provided consent, while adolescents 18–19 years provided informed consent and did not require parental consent. All of the data collection processes were approved by the National Center for Health Statistics Research Ethics Review Board (protocol #2005–06 for years 2007–2010, and protocol #2011–17 for years 2011–2016).

### Measures

#### Waist circumference (WC) and waist-to-height ratio (WHtR)

Anthropometric measures were collected in the Mobile Examination Center (MEC) by trained health technicians. Height (cm) and WC (cm) measurements were collected using standard protocols and equipment [20]. Age and sex-specific WC percentiles were calculated as previously done in other studies [11] and ≥ 90th percentile was considered high risk [21]. Lastly, WHtR was calculated by taking WC (cm) and dividing it by height (cm). A WHtR of ≥0.5 was considered high risk [11]. Adolescents with high-risk WC or WHtR were considered to have abdominal obesity.

#### Sociodemographic characteristics

The demographic questionnaire was administered in the home by trained interviewers. Adolescents aged 16–19 were interviewed directly, whereas those aged 12–15 had a proxy to respond to questions when the child was unable to answer. Adolescents self-reported their sex (male or female) and race/ethnicity. Race/ethnicity categories reported in this study included non-Hispanic (NH) white, NH black, Mexican American, other Hispanics, and other race/multi-racial. Starting in 2011–2012 survey cycle, the NH Asian category was added to the list of NHANES racial/ethnic categories. For this study, NH Asian was included in the other race/multi-racial group in order to maintain consistency throughout the five data cycles.

Two measures are used as proxies for household SES. The first is the education level of the household reference person. This data was collected and was categorized as follows: high school/general education diploma (GED) or less (included those with <9th grade, 9-11th grade education, high school/GED or equivalent degrees), some college (those with some college or Associate degrees), and college graduates or above. The household reference person is the person who owns or rents the house and is most likely the parent. Thus, this variable will be henceforth referred to as parental education level. The second measure is the poverty to income ratio (PIR), which was calculated by dividing family income by the poverty threshold specific to the survey year, also accounting for family size. The PIR falls within three categories; low-income (PIR ≤1.3), middle-income (PIR > 1.3–3.5), and high-income (PIR > 3.5) [17].

#### Physical activity

Adolescents aged 12 to 15 years completed the physical activity questionnaire at the MEC, whereas adolescents 16 years and older completed the questionnaire at home prior to visiting the MEC. In this study, physical activity level (PAL) was assessed by two questions, as previously done in another study [22]; “In a typical week do you do any vigorous-intensity sports, fitness, or recreational activities that cause large increases in breathing or heart rate like running or basketball for at least 10 minutes continuously?”, and “In a typical week, do you do any moderate-intensity sports, fitness, or recreational activities that cause a small increase in breathing or heart rate such as brisk walking, bicycling, swimming, or volleyball for at least 10 minutes continuously?”. Adolescents who reported ‘yes’ to both questions were categorized as having a high PAL, those who reported ‘yes’ to only one question were considered to have a medium PAL, and those who said no to both questions were considered to have a low PAL.

#### Sedentary activity

Sedentary activity was asked during the physical activity questionnaire and was analyzed from the question “How much time do you usually spend sitting on a typical day?”, which may include time spent sitting at a desk, traveling in a car or bus, reading, playing cards, watching television or using a computer. The amount of sedentary time was analyzed as a continuous variable and reported in minutes/day.

### Statistical analyses

Survey weights, strata, and clustering variables were used to account for the complex, multistage probability sampling design used in NHANES, according to the analytic guidelines released by National Center for Health Statistics and CDC [23, 24]. Examination weights were used to create 10-year weights (i.e., 5 cycles) for all analyses. Continuous variables were described using weighted means and categorical variables were summarized with weighted proportions. Standard errors were estimated with Taylor series linearization. The prevalence of high-risk WC and WHtR by survey year and sociodemographic characteristics categories were calculated as percentages with 95% confidence interval (CI). A logistic regression model was conducted to analyze trend of high-risk WC and WHtR across survey years.

Associations between adiposity measures and sociodemographic characteristics (sex, race/ethnicity, parental education level, and PIR) were analyzed using multiple logistic regression models, adjusted for all sociodemographic characteristics, along with age, PAL and sedentary activity. Multicollinearity was assessed by calculating the variance inflation factor. No variables were found to be collinear between one another (VIF ~ 1 for all variables). Significance level was set at *p* ≤ 0.05. For covariates with multiple categories, odds ratios of abdominal obesity outcomes between the different categories were calculated, adjusted using a Bonferroni correction. *P* values for overall trend for parental education level and PIR were also calculated. All analyses were conducted in STATA 15 (StataCorp, College Station, TX).

## Results

Unweighted sample size and weighted means and proportions for individual and household characteristics of the study sample (*n* = 4712) are presented in Table 1. The weighted mean age of the sampled adolescents was 15.5 years. About half of the adolescents were male (50.9%) and half were female (49.1%). The majority of the adolescents were NH white (58.6%), followed by NH black (13.9%), Mexican American (13.1%), other race/multi-racial (7.7%) and other Hispanic (6.7%). About 41.0% of the adolescents’ parent had a high school degree or less, while 31.9% reported to have some college education, and 27.1% reported to be college graduates. Approximately one-third (30.5%) of the adolescents reside in a low-income household (PIR ≤1.3), 37.4% reside in a middle-income household (PIR > 1.3–3.5), and 32.2% reside in a high-income household (PIR > 3.5). Based on the physical activity categories, 18.9% of adolescents had low PAL, 43.1% had medium PAL, and 38.0% have high PAL. On average, adolescents spend 484.8 min (a little over 8 h) of sedentary activities each day. About 18% of adolescents had high-risk WC (≥90th percentile) and 35.5% of adolescents had high-risk WHtR (≥0.5).

**Table 1:** Individual and household characteristics of adolescents (12–19 years) in NHANES 2007–2016 with examination data (*n* = 4712)

Characteristics	Unweighted N^**a**^	Weighted % (SE)^**a**^
Age, mean (SD)	15.47 (1.98)	15.49 (0.04)
Sex		
Male	2433	50.9% (0.92)
Female	2279	49.1% (0.92)
Race/ethnicity		
NH White	1390	58.6% (2.15)
NH Black	1171	13.9% (1.18)
Mexican American	1043	13.1% (1.27)
Other Hispanic	538	6.7% (0.70)
Other race/multi-racial	570	7.7% (0.64)
Parental education^b^		
High school/GED or less	2339	41.0% (1.71)
Some college	1404	31.9% (1.16)
College graduate	969	27.1% (1.57)
Poverty to income ratio^b^		
Low-income (PIR ≤ 1.3)	1996	30.5% (1.52)
Middle-income (PIR > 1.3–3.5)	1683	37.4% (1.38)
High-income (PIR > 3.5)	1033	32.2% (1.67)
Physical activity level^b^		
Low	1008	18.9% (0.75)
Medium	2074	43.1% (1.00)
High	1630	38.0% (1.06)
Sedentary activity minutes (mean (SD))	485.76 (174.47)	484.8 (4.38)
High-risk waist circumference^b^	8555	17.7% (0.84)
High-risk waist-to-height ratio^c^	1785	35.5% (1.03)

*NHANES* National Health and Nutrition Examination Survey, *BMI* Body mass index, *SE* Standard error, *SD* Standard deviation, *GED* General education diploma, *PIR* Poverty to income ratio

^a^Unweighted N (%) represent raw data, weighted % (SE) used 2007–2016 survey weights. Units are as specified unless otherwise stated

^b^High-risk waist circumference defined as greater than or equal to the gender- and age-specific 90th percentile

^c^High-risk waist-to-height ratio defined as ≥0.5

The prevalence of high-risk WC and high-risk WHtR are presented across survey cycles and sociodemographic characteristics in Table 2. The prevalence of high-risk WC ranged from 14.1 to 22.8% and from 30.7 to 49.8% for high-risk WHtR across the race/ethnicity categories. The prevalence of high-risk WC and WHtR are lower with higher levels of parental education and PIR. There were no significant trends in prevalence of high-risk WC and high-risk WHtR over survey years.

**Table 2:** Prevalence of high-risk waist circumference and high-risk waist-to-height ratio in adolescents by sociodemographic characteristics (*n* = 4712)

	Prevalence, % (95% CI)	
	High-risk waist circumference (≥90th percentile)	High-risk waist-to-height ratio (≥0.5)
Survey year		
2007–08	17.5 (14.5–20.9)	34.7 (30.5–39.1)
2009–10	15.2 (13.2–17.4)	31.3 (28.4–34.4)
2011–12	18.1 (14.2–22.7)	37.0 (33.9–40.2)
2013–14	18.1 (14.8–22.1)	36.4 (30.9–42.2)
2015–16	19.3 (15.0–24.6)	37.6 (32.1–43.5)
Sex		
Male	16.8 (14.9–18.9)	30.8 (28.3–33.5)
Female	18.6 (16.4–21.0)	40.2 (37.4–43.1)
Race/ethnicity		
NH White	16.6 (14.4–19.0)	32.0 (29.0–35.1)
NH Black	18.8 (16.1–21.7)	35.4 (32.1–38.9)
Mexican American	22.8 (20.2–25.7)	49.8 (46.5–53.0)
Other Hispanic	18.9 (15.1–23.3)	43.5 (39.1–48.0)
Other/multi-racial	14.1 (10.6–18.4)	30.7 (25.6–36.3)
Parental education		
High school/GED or less	21.3 (18.7–24.2)	41.3 (38.8–43.8)
Some college	18.6 (16.5–21.0)	37.2 (33.8–40.7)
College graduate	11.0 (8.7–13.9)	24.5 (21.1–28.3)
Poverty to income ratio		
Low-income (PIR ≤ 1.3)	21.6 (19.2–24.1)	43.9 (41.2–46.6)
Middle-income (PIR > 1.3–3.5)	18.8 (16.2–21.6)	37.6 (34.1–41.3)
High-income (PIR > 3.5)	12.7 (10.3–15.6)	24.9 (21.7–28.5)

*NHANES* National Health and Nutrition Examination Survey, *CI* Confidence interval, *NH* Non-Hispanic *GED* General education diploma, *PIR* Poverty to income ratio

Associations between sociodemographic characteristics and high-risk WC and WHtR are presented in Table 3. There was a significant linear trend in high-risk WC by parental education level (*p* = 0.004). When the parent had a college degree, adolescents were less likely to have a high-risk WC (OR = 0.57, 95%CI = 0.37–0.87, *p* = 0.006) than when parent had a high school degree or less. Adolescents whose parent had a college degree were also significantly less likely to have high-risk WC compared to those whose parent had some college education (OR = 0.62, 95%CI = 0.43–0.89, *p* = 0.006). While there were no significant differences in the likelihood of having high-risk WC by household income categories, there was overall, a significant linear trend of lower odds for high-risk WC as household PIR increases (*p* = 0.04) (Table 3). There was no association between other sociodemographic characteristics and high-risk WC.

**Table 3:** Odds ratios of high-risk waist circumference and high-risk waist-to-height ratio in adolescents by sociodemographic characteristics (*n* = 4712)

	Odds ratios (95% CI)	
	High-risk waist circumference (≥90th percentile)	High-risk waist-to-height ratio (≥0.5)
Sex		
Male	Ref^a^	Ref^a^
Female	1.05 (0.87–1.28)^a^	1.46 (1.23–1.72)^b^
Race and ethnicity		
NH white	Ref^a^	Ref ^a^
NH black	0.96 (0.67–1.38)^a^	0.96 (0.69–1.32)^a^
Mexican American	1.15 (0.80–1.63)^a^	1.66 (1.24–2.20)^b^
Other Hispanic	0.94 (0.60–1.48)^a^	1.33 (0.95–1.87)^ab^
Other/multi-racial	0.76 (0.45–1.28)^a^	0.90 (0.58–1.41)^a^
Parental education		
High school/GED or less	Ref ^a^	Ref ^a^
Some college	0.92 (0.66–1.28)^a^	1.03 (0.80–1.33)^a^
College graduate	0.57 (0.37–0.87)^b^	0.70 (0.51–0.97)^b^
*P* value^c^	0.004	0.01
Poverty to income ratio		
Low-income (PIR ≤ 1.3)	Ref^a^	Ref^a^
Middle-income (PIR > 1.3–3.5)	0.92 (0.67–1.26)^a^	0.86 (0.66–1.11)^a^
High-income (PIR > 3.5)	0.72 (0.48–1.07)^a^	0.58 (0.43–0.78)^b^
*P* value^c^	0.04	< 0.001

*NHANES* National Health and Nutrition Examination Survey, *CI* Confidence interval, *Ref* Reference category, *NH* Non-Hispanic, *GED* General education diploma

Multiple logistic regression models were adjusted for age, physical activity level, and sedentary activity. Values are odds ratios (95% CI) between a level of a variable compared to the reference category

^a, b^Values with different subscripts are significantly different at *P* ≤ 0.05 within each variable after Bonferroni correction

^c^*P* value for trend used when categories within a variable is ordered, significance level is set at *p* ≤ 0.05

Regarding WHtR, females were more likely to have a high-risk WHtR compared to males (OR = 1.46, 95%CI = 1.23–1.72, *p* < 0.001). Mexican American adolescents had higher odds for high-risk WHtR compared to NH white (OR = 1.66, 95%CI = 1.24–2.20, p < 0.001), NH black (OR = 1.73, 95%CI = 1.26–2.36, *p* < 0.001) and other race/multi-racial adolescents (OR = 1.84, 95%CI = 1.21–2.80, *p* = 0.001). There were significant linear trends in high-risk WHtR by parental education level (*p* = 0.01) and household PIR (*p* < 0.001). Adolescents with parent who had a college degree had significantly lower odds for high-risk WHtR than adolescents whose parent had some college education (OR = 0.68, 95%CI = 0.49–0.93, *p* = 0.01) and had a high school degree or less (OR = 0.70, 95%CI = 0.51–0.97, *p* = 0.03). For PIR, odds for high-risk WHtR was significantly lower in adolescents living in high-income households than in adolescents living in low-income households (OR = 0.58, 95%CI = 0.43–0.78, *p* < 0.001) and adolescents residing in middle-income household (OR = 0.68, 95%CI = 0.48–0.96, *p* = 0.022).

## Discussion

In the U.S., a large percentage of adolescents have abdominal obesity. However, there is limited information on the association between various sociodemographic characteristics and WC, as well as WHtR, among U.S. adolescents. Understanding sociodemographic disparities in abdominal obesity is important as high WC and WHtR in adolescents have been shown to predict future adverse health outcomes [12–16]. This study described the prevalence of WC and WHtR, as well as associations between several sociodemographic characteristics and WC and WHtR, among a nationally representative sample of U.S. adolescents.

In the current study, female adolescents had greater odds for a high-risk WHtR compared to males, but there was no association between sex and WC. By contrast, Xi et al. (2014) found female children and adolescents (2–19 years) to be at greater risk for both WHtR and WC than males [11]. This discrepancy could be due to the age differences in the sample used by the current study (12–19 years) and that of Xi et al.’s. (2–19 years). Additionally, the current study used data from 2007 to 2016, whereas Xi et al.’s study used data from 2003 to 2012. Another possible explanation for the lack of association between sex and WC is that fat distribution in male adolescents becomes more centered in the abdominal region during late puberty (age 14), whereas for females, fat distribution remains in the gynoid pattern throughout puberty [25]. Moreover, there is evidence that male adolescents typically exceed female adolescents in height by age 14 and they continue to grow until they reach their peak height [26]. Due to these factors, male adolescents’ WHtR on average, would be smaller than that for female adolescents given similar WC. These developmental changes may explain why differences in WHtR by sex, but not WC, were observed in the current study.

Mexican American adolescents are significantly more likely to have high-risk WHtR, compared to NH white and black, and other race/multi-racial adolescents. Higher prevalence of abdominal obesity as measured by WHtR in Mexican American adolescents compared to NH white adolescents has been reported previously using NHANES data from 2003 to 2012 [11]. Although, comparisons of prevalence of high-risk WHtR between Mexican American adolescents and adolescents of other race/ethnic identities have never been evaluated.

It is known that WHtR distinguishes between lean mass and fat and has been suggested to be a better predictor of adiposity compared to BMI [27]. A couple of studies have also argued that BMI is less reliable when used to examine differences in adiposity between racial and ethnic groups since the relationship between body fat composition and BMI differs across ethnic groups [28, 29]. Thus, associations between sociodemographic characteristics with abdominal obesity and BMI were not consistent. In the current study, likelihood of having high-risk WHtR in NH black adolescents were not significantly different than that in adolescents of any other race/ethnic identities. On the other hand, studies focusing on excess BMI in adolescents commonly report that obesity prevalence in NH black adolescents are significantly higher than in NH white adolescents [6, 7].

In the current study, there were no significant differences in likelihood of having high-risk WC by race/ethnicity. In contrast, previous studies that analyzed NHANES data from 1988 to 2002 and 2003 to 2012 found those who identified as Mexican Americans or Hispanics were more likely to have high-risk WC compared to NH white and NH black youth [11, 30]. The data used in the current study includes the most recent NHANES cycle (2015–2016 cycle). Thus, it is possible that associations between race/ethnicity and high-risk WC have changed over the years. Additionally, unlike the previous studies, the current study used Bonferroni correction to adjust for multiple comparisons within the different race/ethnic categories which resulted in larger confidence intervals for the odds ratios calculated in the models. Another study, by Tybor et al. (2010), found NH black female adolescents having significantly higher WC than white female adolescents [31]. Tybor et al., however, focused only on female adolescents rather than both male and female adolescents, as done in the current study [31].

To the authors’ knowledge, this is the first study to report that both high-risk WC and WHtR in U.S. adolescents are associated with parental education (a proxy for household SES). For both WC and WHtR, the odds of abdominal obesity were significantly lower in adolescents whose parent are college graduates, compared to parent who have some college education, indicating that higher educational attainment in parent is related to the health status of adolescents. This result is consistent with studies that examined associations between SES and BMI, which have shown that obesity prevalence among U.S. adolescents is inversely associated with parental education level [6, 17]. For example, Ogden and colleagues (2018) found that U.S. children and adolescents (2–19 years) with parent with a high school degree or less were significantly more likely to be obese compared to youth whose parent graduated from college, 22% and 12% respectively [6]. Furthermore, this result also corroborates analyses of the Healthy Lifestyle in Europe by Nutrition in Adolescence (HELENA) study, where parental education level was inversely associated with WC and WHtR [32]. It has been suggested that education level is associated with dietary intake/quality as those with higher education may have access to greater nutrition knowledge, which also better allows for incorporation of such knowledge into their dietary habits [33].

In the current study, likelihood of high-risk WC did not differ significantly across PIR categories. This result aligns with analyses of the HELENA study in Europe which found no association between affluence (measured by Family Affluence Scale) and WC [32]. By contrast, this study found that odds of having high-risk WHtR is significantly lower in adolescents living in high-income households (PIR > 3.5) compared to adolescents living in middle-income and low-income households. Similarly, Ali et al. (2011), using data from NHANES 1998–2008, reported that prevalence of high-risk WHtR in children and adolescents was inversely related to household income status [34]. Other NHANES studies have also found that BMI is significantly lower in adolescents living in high-income households compared to low-income households [17, 18].

While parental education level and PIR are both indicators of SES, this study found that the association between the two measures and abdominal obesity are different. Previous research has shown parental education level may be a better indicator for an individual’s weight status and other chronic conditions [17, 35]. Despite the differences, this study revealed a general trend where adolescents from low SES households have a greater risk for abdominal obesity. One possible explanation of the relationship between SES and abdominal obesity is that individuals residing in higher income neighborhoods may have more access to recreation and pedestrian/biking facilities, and thus higher levels of physical activity compared to individuals in low-income neighborhoods [36]. Individuals living in higher-income areas also have more access to healthier foods [37]. In fact, neighborhoods’ walkability, shopping facilities, and existence of parks/playgrounds and SES have been found to be associated with WC in children and adolescents with severe obesity [38].

This study is not without limitations. In NHANES, the sample size for some demographic subgroups are small, which may result in inaccurate estimates. To overcome this, the study combined multiple data cycles (2007–2016) to obtain a higher sample size for specific demographic subgroups, such as other Hispanic and other race/multi-racial adolescents. They were included in the study as separate categories since there is little data on the adiposity status for adolescents in these groups otherwise. The study used cross-sectional data, thus no conclusions regarding causality between sociodemographic characteristics and abdominal obesity can be made. While multiple statistical tests were conducted, associations reported in the study were corrected to account for multiple comparisons, increasing the robustness of the statistical analyses.

Categories for PAL used may not accurately estimate how active an adolescent is as it did not distinguish the types and actual duration of physical activity that adolescents engage in. Although the Metabolic Equivalent of Task (MET) scores can be used to measure PAL [39], information needed to calculate METs score had a significant proportion of missing data and was deemed insufficient for analyses. This study did not include information on nutritional intake and pubertal development which could affect adiposity and fat distribution in adolescents. Additionally, pregnant adolescents were not excluded from the study even though pregnancy status would impact adiposity status. Pregnancy status in NHANES 2007–16 were not released for individuals below the age of 20 due to disclosure risks [40]. However, according to NHANES data documentation, the proportion of pregnant adolescents aged 12–19 years old were estimated to be very low (1–2%) [40], thus the inclusion of pregnant adolescents likely had little impact on the results.

Despite these limitations, this study has several noteworthy strengths. As discussed above, the current study is one of the first studies to explore the association of household SES and both WC and WHtR among U.S. adolescents in recent years. Moreover, data from NHANES is nationally representative, thus results from this study are generalizable to U.S. adolescents. Anthropometrics measurements were collected by trained staff using standard protocols [20] and thus, estimates of WC and WHtR presented in the study were less prone to measurement error.

## Conclusion

This study addresses a critical gap in the literature by revealing that sociodemographic characteristics (i.e., sex, racial/ethnic identities, parental education level, and PIR) are associated with high-risk WC and WHtR among U.S. adolescents. Many studies that have examined associations between sociodemographic characteristics and adiposity use BMI as a measure of adiposity [6, 8, 41]. However, assessing only BMI may not fully capture the burden of adiposity in adolescents. This is important as disparities in chronic disease prevalence persist into adulthood; with rates reported to be significantly higher in racial/ethnic minority and low-income adults [42–44]. Future interventions for adolescents should prioritize those who are most at-risk for abdominal obesity, namely female adolescents, as well as adolescents from racial/ethnic minority and low socioeconomic status backgrounds. Additionally, such interventions should consider including changes in WC and WHtR as a measure of impact of these interventions, rather than just BMI.

## Data Availability

All data presented in the manuscript are publicly available through the Centers for Disease Control and Prevention site at https://wwwn.cdc.gov/nchs/nhanes/default.aspx [45]. Additionally, STATA software was used. Operating system: Platform independent, requirements: STATAIC 14 or higher, license: individual, any restrictions to use by non-academics: license needed.

## Abbreviations

WC: Waist circumference
WHtR: Waist-to-height ratio
BMI: Body mass index
CDC: Centers for Disease Control and Prevention
SES: Socioeconomic status
NHANES: National Health and Nutrition Examination Survey
NH: Non-Hispanic
GED: General education diploma
PIR: Poverty to income ratio
MEC: Mobile examination center
PAL: Physical activity level
CI: Confidence interval
SD: Standard deviation
SE: Standard error
HELENA: Healthy Lifestyle in Europe by Nutrition in Adolescence
MET: Metabolic Equivalent of Task
